# The Allergic Rhinitis - Clinical Investigator Collaborative (AR-CIC) - optimizing the Nasal Allergen Challenge (NAC) model

**DOI:** 10.1186/1710-1492-10-S2-A62

**Published:** 2014-12-18

**Authors:** Mena Soliman, Jenny Thiele, Lisa Steacy, Marie-Eve Boulay, Angela Hillaby, Susan Waserman, Paul Keith, Harissios Vliagoftis, Louis-Philippe Boulet, Helen Neighbour, Anne K Ellis

**Affiliations:** 1 Departments of Medicine and Biomedical & Molecular Science, Queen’s University, Kingston, Ontario, K7L 3N6, Canada; 2 Allergy Research Unit, Kingston General Hospital, Kingston, Ontario, K7L 2V7, Canada; 3 Institut Universitaire de Cardiologie et de Pneumologie de Quebec, Quebec City, Quebec, G1V 4G5, Canada; 4 Pulmonary Research Group, University of Alberta, Edmonton, Alberta, T6G 2R7, Canada; 5 Department of Medicine, McMaster University, Hamilton, Ontario, L8S 4K1, Canada; 6 Firestone Institute for Respiratory Health, McMaster University, Hamilton, Ontario, L8N 4A6, Canada

## Background

We sought to optimize the Nasal Allergen Challenge (NAC) model to ensure reliability and repeatability of results by modifying the qualifying criteria and allergen concentration during the challenge.

## Methods

20 Allergic Rhinitis (AR) participants underwent NAC to determine the concentration at which a Total Nasal Symptom Score (TNSS) of 10/12 OR a Peak Nasal Inspiratory Flow (PNIF) reduction of 50 % was achieved. 4-fold increases in allergen concentration were administered every 15 minutes until qualification criteria were met. The Qualifying Allergen Concentration (QAC) reached was used as a single challenge dose at the subsequent NAC visit. 10 additional ragweed allergic and 4 non-allergic participants were qualified at a TNSS of 8/12 AND a PNIF reduction of 50%. Cumulative Allergen Concentration (CAC) of all incremental doses was used during the subsequent NAC visit. Participants recorded TNSS and PNIF at baseline, 15 minutes, 30 minutes, 1 hour and hourly afterwards up to 12 hours post-challenge during the NAC visit.

## Results

QAC study participants qualifying only based on PNIF reduction had significantly lower TNSS scores than those qualifying on TNSS only or TNSS+PNIF (p<0.01). Participants in both studies’ NAC visit reached peak TNSS at 15 minutes post-challenge followed by a gradual symptom decline, while the “PNIF only” group had significantly lower TNSS compared to others. All 3 groups experienced a decline in peak TNSS following NAC compared to screening, although groups qualifying on TNSS and TNSS+PNIF maintained their PNIF scores.

## Conclusion

The NAC model is well-suited to study AR symptoms. TNSS and PNIF are complementary and must be integrated in the qualifying criteria. Further protocol modifications, such as with multiple allergen challenges during the NAC visit, may produce even more repeatable results. Through optimizing the NAC protocol, the model achieves reproducible results and becomes more reliable; suitable for testing new medications in clinical trials.

